# Pragmatic Home-Based Exercise after Total Hip Arthroplasty - Silkeborg: Protocol for a prospective cohort study (PHETHAS-1)

**DOI:** 10.12688/f1000research.19570.1

**Published:** 2019-10-14

**Authors:** Lone Ramer Mikkelsen, Merete Nørgaard Madsen, Michael Skovdal Rathleff, Kristian Thorborg, Camilla Blach Rossen, Thomas Kallemose, Thomas Bandholm

**Affiliations:** 1 Elective Surgery Centre, Silkeborg Regional Hospital, Silkeborg, Denmark; 2 Department of Clinical Medicine, Aarhus University, Aarhus, Denmark; 3 Center for General Practice, Department of Clinical Medicine, Aalborg University, Aalborg, Denmark; 4 Sports Orthopedic Research Center-Copenhagen, Department of Orthopaedic Surgery, Copenhagen University Hospital, Amager and Hvidovre, Hvidovre, Denmark; 5 Physical Medicine & Rehabilitation Research - Copenhagen (PMR-C), Department of Occupational and Physical Therapy, Copenhagen University Hospital, Amager and Hvidovre, Hvidovre, Denmark; 6 Clinical Research Centre, Copenhagen University Hospital, Amager and Hvidovre, Hvidovre, Denmark; 7 Department of Physical and Occupational Therapy, Copenhagen University Hospital, Amager and Hvidovre, Hvidovre, Denmark

**Keywords:** Total Hip Arthroplasty, Rehabilitation, Exercise therapy, Dose-response, Strength training

## Abstract

**Introduction: **Rehabilitation exercises are offered to patients after total hip arthroplasty (THA); however, the effectiveness and optimal type and dose of exercise remains unknown. The primary objective of this trial is to indicate the preliminary efficacy of home-based rehabilitation using elastic band exercise on performance-based function after THA, based on the relationship between the performed exercise dose and the change in performance-based function (gait speed) from 3 (start of intervention) to 10 weeks (end of intervention) after surgery. The secondary objective is to investigate if a dose-response relationship exists between the performed exercise dose and changes in: hip-related disability, lower-extremity functional performance, and hip muscle strength

**Methods:** In this prospective cohort study, patients scheduled for THA will be consecutively included until 88 have completed the intervention period from 3 to 10 weeks postoperatively. Participants perform the standard rehabilitation program with elastic band exercises. Exercise dose (exposure) will be objectively quantified using a sensor attached to the elastic band. The primary outcome is gait speed measured by the 40-m fast-paced walk test. Secondary outcomes include: patient reported hip disability (Hip disability and Osteoarthritis Outcome Score (HOOS)), hip muscle strength (hand-held dynamometry) and lower extremity function (30-s chair stand test).

**Discussion: **This trial will add knowledge concerning the relationship between performed exercise dose and post-operative outcomes after THA. The protocol paper describes the study design and methods in detail, including the statistical analysis plan.

**Trial registration: **Pre-registered on March 27, 2017 at ClinicalTrails.gov (ID: NCT03109821).

## Introduction

Total hip arthroplasty (THA) ^1^ is offered to patients with end-stage hip osteoarthritis to reduce pain and improve function ^1^. Muscle strength and functional performance, such as walking ability, are substantially reduced early after THA ^2– 5^; this is why postoperative rehabilitation is offered throughout the municipalities in Denmark. In some municipalities, this is organized as outpatient supervised rehabilitation, whereas in other municipalities, patients receive an initial instruction and perform rehabilitation exercise in their own homes without supervision. In Central Denmark Region (place of this trial), the current predominant clinical practice is home-based rehabilitation for most patients.

Systematic reviews with meta-analyses show that supervised, outpatient rehabilitation exercise is not superior to home-based exercise for performance-based or self-reported function outcomes ^6, 7^. It has also been difficult to demonstrate clear superiority with relevant effect size of one type of rehabilitation exercise over another for performance-based or self-reported function outcomes ^8, 9^. There is, however, some evidence to indicate that rehabilitation exercise may be superior to no or very little rehabilitation exercise for selected muscle-strength, gait, and function outcomes after THA ^6, 9, 10^. It suggests that a dose-response relationship exists for post-operative rehabilitation exercise and recovery after THA.

To be able to investigate a dose-response relationship for post-operative rehabilitation exercise and recovery after THA, objective measures that capture compliance to home-based exercise are needed ^11^. In recent work ^12– 14^, we have validated a measure to monitor compliance to home-based exercise in healthy subjects (an in-built sensor attached to an elastic exercise band), and started using it in clinical populations for intervention research ^15– 18^. With the PHETHAS-1 trial, we want to use this sensor technology to investigate if a dose-response relationship exists for home-based rehabilitation exercise and recovery after THA, using a prospective cohort study design. By using this technology, we will be able to not only investigate a dose-response relationship on the recovery associated with exercise, but also investigate the preliminary efficacy of home based, rehabilitation exercise after THA. This can be achieved by comparing participants with the least exercise compliance to those with the most. This will indicate whether home-based, rehabilitation exercise “works” better than no or very little rehabilitation exercise, although not a randomized comparison. It will help inform a subsequent large-scale, confirmatory, randomized trial investigating the efficacy of rehabilitation exercise after THA when compared to no or very minimal rehabilitation exercise.

### Objectives

The primary objective is to indicate the preliminary efficacy of home-based rehabilitation using elastic band exercise on performance-based function after THA, based on the relationship between the performed exercise dose and the change in performance-based function (gait speed measured by 40-m fast-paced walk test) from 3 (start of intervention) to 10 weeks (end of intervention) after surgery.

The secondary objective is to investigate if a dose-response relationship exists between the performed exercise dose and changes in: hip-related disability, lower-extremity functional performance, and hip muscle strength.

## Methods

### Study design

The study is a pragmatic, single-center, prospective cohort study (single cohort) conducted in Silkeborg, Denmark. By pragmatic study we mean that the study reflects real life for the involved trial stakeholders. In this study this is for instance reflected by the type and dose of exercise which reflects our current practice. Outcome assessments will be performed at 3 (start of home-based strengthening exercise) and 10 weeks (after 7 weeks of home-based strengthening exercise) after surgery. Furthermore, patient-reported outcome measures will be collected pre-surgery (see the participant timeline in Table 1). It is the aim that all outcome assessments will be performed by three physiotherapists who have been thoroughly trained in performing the outcome assessments. The data collection methods, trial logistics and the intervention have been tested in a pilot study including 10 patients and adjustments have been made accordingly. The study will adhere methodologically to the STROBE guideline for prospective cohort studies and the CONSORT statement.

**Table 1.** Participant timeline.

	Study period			
Time point	Admission	Baseline	Intervention	Follow up
	Pre or post surgery	3 week visit at the hospital	Week 3–10 post THA	10 week visit at the hospital
Enrollment				
Eligibility screen	X (pre)			
Informed consent	X (pre)			
Interventions				
Unloaded exercise	X (post) →			
Strengthening exercise		Exercise instruction	X	
Assessments				
Elastic band sensor (BandCizer)			X	
40-m fast-paced walk test		X		X
HOOS *	X (pre)	X		X
30-s chair stand test		X		X
Hip muscle strength		X		X
Pain: VAS ** at rest before + after exercise			X	
Self-reported additional exercises			X	
Self-efficacy	X (pre)	X		
Physical activity (ActivPal)			X (7 days data collection)	
Adverse events		X		X
Motivation to exercise as prescribed		X		
Evaluation of prescribed exercises				X
Change in hip problems				X
Perception of result after surgery				X

* HOOS: Hip disability and Osteoarthritis Outcome Score ** VAS: Visual Analogue Scale

### Study setting

All participants will be included from the Elective Surgery Centre at the public hospital, Silkeborg Regional Hospital. Exercise instruction as well as blinded outcome assessments will be performed by physiotherapists from Elective Surgery Centre. The physiotherapists are members of the staff of physiotherapists at Elective Surgery Centre and all have at least 6 months of experience working with THA.

### Participants

Participants will be included by consecutive sampling. The inclusion criteria are: age above 18 years, scheduled for a primary THA at the Elective Surgery Centre due to osteoarthritis and able to understand written and spoken Danish. The exclusion criterion is: referral to supervised rehabilitation in the municipality (instead of the home-based rehabilitation exercise-program in the present study).

### Intervention

We define rehabilitation exercise as: “A regimen or plan of physical activities designed and prescribed for specific therapeutic goals. Its purpose is to restore normal musculoskeletal function or to reduce pain caused by diseases or injuries." The exercise intervention in the present study reflects the standard rehabilitation exercise practice at Elective Surgery Centre; hence, a pragmatic approach is used. The exercises in the present trial are comparable to the control intervention in a previous study from our department where we compared usual care (home-based exercise using elastic band resistance) to supervised progressive resistance training in machines and found comparable effects ^19^. During a short hospital stay (typically discharge on the day after surgery), all patients are instructed in an exercise program of unloaded exercises (not part of the intervention studied) to be performed at home during the initial 3 postoperative weeks until their scheduled follow up visit at the hospital. At this visit (3 weeks after surgery), and after the outcome assessment, the participants will receive a thorough instruction in the strengthening exercises that they are instructed to perform without supervision in their own homes the following 7 weeks. The instruction is conducted one-to-one by physiotherapists using approximately 20 minutes per participant and supported by an instruction booklet with written and illustrated exercise descriptions. The strengthening exercises included are: hip abduction, flexion and extension with elastic band resistance and sit-to-stand. The prescribed training load will be two sets with repetitions to contraction failure (neuromuscular fatigue) and a relative load of 10 to 20 repetition maximum (RM), performed every second day (3–4 times a week). The strengthening exercises are supplemented with daily stretching of hip flexor muscles and balance exercise (one-legged stance). Exercise compliance for the strengthening exercises will be monitored objectively (see *Outcomes* section). No efforts will be made to increase compliance beyond normal practice (e.g. SMS encouragements, or likewise), because we intent to measure actual, uninfluenced compliance as close to daily practice as possible. The patients are recommended to perform daily walking with increasing distance during their rehabilitation. They are advised to gradually increase their general activity level after the operation to comply with the recommendations on physical activity from the Danish Health and Medicines Authority (≥30 minutes/day of physical activity with moderate intensity + 20 minutes twice a week of physical activity with high intensity). Furthermore, they will be given instructions on how to handle pain during exercises and recreational activities (the pain management guide is available as *Extended data*) ^20^. To reinforce similar treatment administration, face-to-face meetings among the participating physiotherapists will be held per need to discuss issues experienced in the clinic. The exercise intervention is described in detail according to the exercise-specific Consensus on Exercise Reporting Template (CERT) ^21^ (A completed CERT checklist is available as *Extended data*) ^20^, supplemented with the full set of strength training descriptors as suggested by Toigo and Boutellier ( Table 2) ^22^. Finally, the exercise intervention is described according to the Template for Intervention Description and Replication (TIDieR) checklist, which is a generic intervention-description template (a completed TIDieR checklist is available as *Extended data*) ^20, 23^.

**Table 2.** Strengthening exercise descriptors ^22^.

	Hip abduction	Hip flexion	Hip extension	Sit-to-stand
Load	15 RM *, acceptable interval: 10–20 RM	15 RM, acceptable interval: 10–20 RM	15 RM, acceptable interval: 10–20 RM	15 RM, acceptable interval: 10–20 RM
Repetitions	10–20	10–20	10–20	10–20
Set per session	Week 1: 1 set (both legs) Week 2–7: 2 sets (both legs)	Week 1: 1 set (both legs) Week 2–7: 2 sets (both legs)	Week 1: 1 set (both legs) Week 2–7: 2 sets (both legs)	Week 1: 1 set Week 2–7: 2 sets
Rest between sets	Active rest while exercising opposite leg	Active rest while exercising opposite leg	Active rest while exercising opposite leg	1–3 minutes
Sessions per week	3–4 (every second day)	3–4 (every second day)	3–4 (every second day)	3–4 (every second day)
Duration of training period	7 weeks	7 weeks	7 weeks	7 weeks
Contraction modes	2 seconds concentric, 1 second isometric, 2 seconds eccentric	2 seconds concentric, 1 second isometric, 2 seconds eccentric	2 seconds concentric, 1 second isometric, 2 seconds eccentric	2 seconds concentric, 1 second isometric, 2 seconds eccentric
Rest between repetitions	0 sec, possible load relieve with one step between reps if needed	0 sec, possible load relieve with one step between reps if needed	0 sec, possible load relieve with one step between reps if needed	0 sec
Time under tension	150 sec/exercise/session at 15 RM	150 sec/exercise/session at 15 RM	150 sec/exercise/session at 15 RM	150 sec/exercise/session at 15 RM
Contraction failure in each set	Yes. The exercise is progressed (elastic band with higher load) when >20 repetitions are accomplished.	Yes. The exercise is progressed (elastic band with higher load) when >20 repetitions are accomplished.	Yes. The exercise is progressed (elastic band with higher load) when >20 repetitions are accomplished.	Yes. The exercise is progressed (backpack with weights) when >20 repetitions are accomplished.
Range of motion	Maximum possible	Maximum possible	Maximum possible	Approximately from 90 to 0 degrees of hip and knee flexion.
Rest between sessions	48 hours	48 hours	48 hours	48 hours
Anatomical definition of the exercises	Hip abduction is performed in upright standing position with the elastic band looped around both ankles and support by e.g. a solid table. The hip is abducted as much as possible with the toes pointing directly forward and keeping the trunk in upright position.	Hip flexion is performed in upright standing position with the elastic band under the foot of the stance leg and around the ankle of the target leg and support by e.g. a solid table. The target leg is elevated against resistance in a combined hip and knee flexion while keeping the trunk in upright position.	Hip extension is performed in upright standing position with the elastic band looped around both ankles and support by e.g. a solid table. The hip is extended with the ankle flexed to avoid floor contact while keeping the trunk in upright position.	The exercise is performed from standing with equal load on both legs and toes pointing forward. With arms crossed the participants slowly sits down until the chair is just touched and then rises again.

* RM: Repetition Maximum

### Patient information

The participants will be advised to gradually increase their activity level after the operation. Likewise, they will be instructed to gradually progress their exercises during the 7 weeks of training at home according to the described progression model, where the strengthening exercises are performed to failure in each set; when the possible repetitions exceed 20 in two of the three elastic band exercises they should change the elastic band so that a higher loading is possible. The participants are instructed that pain in relation to exercise is normal, and that up to 5 on a numeric rating scale (NRS) during exercise is considered acceptable based on the suggested pain monitoring system by Thomée *et al.* ^24^. However, the pain should decrease within 30 minutes after the exercise session. The participants are advised to contact the hospital if they experience increasing pain or other complications such as swelling or wound problems (the pain management guide is available as *Extended data*) ^20^.

### Outcomes

***Exposure.*** Performed exercise dose will be quantified as the total physiological exercise stimulus (Time under tension summary dose per week) recorded by a sensor (Bandcizer: commercially available from www.bandcizer.com) attached to the elastic exercise band. The sensor automatically switches on and stores data when the elastic exercise band is used ^13, 14^. Furthermore, performed exercise dose will be quantified as the number of days with strengthening exercises being performed.

### Primary outcome

Change in gait speed is chosen to be primary outcome, as walking ability is considered the most important function to improve by patients undergoing THA surgery ^25^. Furthermore, the 40-m fast-paced walk test is part of the core set of functional tests to include in clinical trials in patients with osteoarthritis in hip or knee recommended by OARSI ^26, 27^.

- Change in gait speed
  Measured by the 40-m fast-paced walk test ^26, 27^. Change from 3 to 10 weeks after surgery.

### Secondary outcomes

- Gait speed
  Measured by the 40-m fast-paced walk test ^26, 27^. At 10 weeks after surgery.
- Change in patient-reported function
  Measured by the Activities of Daily Living (ADL) subscale of HOOS ^28^. HOOS is a disease-specific patient-reported outcome measure. Change from 3 to 10 weeks after surgery.
- Change in patient-reported symptoms
  Measured by the symptoms subscale of HOOS ^28^. Change from 3 to 10 weeks after surgery.
- Change in patient-reported pain
  Measured by the pain subscale of HOOS ^28^. Change from 3 to 10 weeks after surgery.
- Change in patient-reported hip related quality of life
  Measured by the quality of life subscale of HOOS ^28^. Change from 3 to 10 weeks after surgery.
- Change in lower extremity function.
  Measured by the 30-s chair stand test ^26, 27^ (The maximal number of rises from a chair within 30 seconds). Change from 3 to 10 weeks after surgery.
- Change in hip abductor muscle strength.
  Test of isometric muscle strength in hip abduction in the operated leg. The hand-held dynamometer Power Track II Commander will be used to assess this using standardized test procedure ^29^. Change from 3 to 10 weeks after surgery.
- Change in hip flexor muscle strength.
  Test of isometric muscle strength in hip flexion in the operated leg. The hand-held dynamometer Power Track II Commander will be used to assess this using standardized test procedure ^29^. Change from 3 to 10 weeks after surgery.

### Other pre-specified outcomes

- Self-efficacy.
  The general self-efficacy scale ^30^ will be used to measure self-efficacy, defined as an individual's belief in his or her capacity to execute behaviors necessary to produce specific performance attainments. At 3 weeks after surgery.
- 24-hour physical activity (mean upright time/day and mean number of steps/day).
  An ActivPAL movement-sensor will be used to measure mean time per day in upright position (standing and walking) based on 7 days of data collection. The sensor will be applied 3 weeks after surgery and used the following week. At 4 weeks after surgery.
- Number of participants with adverse events.
  Number and type of adverse events will be registered by the physiotherapist 3 and 10 weeks after surgery in the following pre-defined categories: Hip dislocation, infection, fracture, wound seepage, acute myocardial infarction, deep venous thrombosis, readmission and other.
- Mean change in pain after each exercise session.
  The visual analogue scale (VAS) will be used to assess pain before and after each exercise session. Data will be summarized as a mean change in pain per exercise session for the entire intervention period. At 10 weeks after surgery.
- Number of pain flares after exercise sessions.
  VAS will be used to assess pain before and after each exercise session. Pain flare is defined as an increase in pain of ≥20 mm ^31^. Data will be summarized, both for the first 14 days of the intervention and for the entire intervention period. At 5 and 10 weeks after surgery.
- Motivation to perform the prescribed exercises.
  The participants will be asked about their motivation to perform the prescribed exercises. A short questionnaire comprising three questions developed for this purpose will be used (the questionnaire is available as *Extended data*) ^20^. The possible responses are ordered in 4 levels of motivation on an ordinal scale. At 3 weeks after surgery.
- Evaluation of the prescribed exercises
  The participants will be asked to evaluate the exercises. A short questionnaire comprising three questions developed for this purpose will be used (the questionnaire is available as *Extended data*) ^20^. The possible responses are ordered in 4 levels on an ordinal scale. At 10 weeks after surgery.

### Changes to outcomes after trial registration

- At June 28, 2017, two outcome measures were added to the study. At 10 weeks after surgery, participants will be asked both to describe their perception of the result after surgery and the change in hip problems (from preoperatively to 10 weeks after surgery). The questions will be phrased as "How would you describe the result of your operation?" with response categories "Excellent", "Very good", "Good", "Fair", "Poor". The second question will be asked as "Overall, how are the problems now in the hip on which you had surgery, compared to before your operation?" with the response categories "Much better", "A little better", "About the same", "A little worse", "Much worse". These two questions have been used as anchor questions to establish patient acceptable symptom state (PASS) and minimal clinically important improvement (MCII) cut-points for patient-reported outcomes – including some subscales of HOOS – 1 year after THA ^32^. We will use these questions to group patients according to their perception of result of the operation and changes in hip problems, as well as for exploratory analysis of PASS and MCII cut-points for HOOS, 10 weeks after surgery.
- In April, 2019, pain flare was added as an outcome measure.
- Categories of adverse events were defined prior to study start, but they were not specifically described in the trial registration. Motivation to perform prescribed exercises was registered as outcome, but although predefined, the three items in the short questionnaire were not specifically described. Evaluation of prescribed exercises was added as outcome prior to study start.
- In April 2019, the secondary objective was added to the primary and pre-specified objective because the primary objective did not clearly outline the secondary analyses of secondary outcomes for the hypothesized dose-response relationship.
- All the changes outlined above occurred before the last participant was included and the study was unblinded (please see “Blinding” below).

### Embedded qualitative study (PHETHAS-2)

In addition to collecting quantitative data, we will also conduct an embedded qualitative study concerning the participants' experience with performing home-based exercise and resuming general physical activities. The aim will be to understand the patients' motivation and barriers related to home-based exercise and general physical activity after THA. The participants will be selected through theoretical sampling ^33^, expectedly a maximum of 20. Participants will be recruited partly from the PHETHAS-1 trial, and partly from the population of standard THA patients not involved in an exercise trial. This is done to elucidate the influence of participating in a trial with extra interventions such as exercise diary, outcome assessments, etc. Semi-structured interviews will be conducted 10 weeks postoperatively using an interview guide (available as *Extended data*) ^20^. This qualitative study is undertaken to refine the home-based intervention for future trials and clinical implementation. The embedded qualitative study will be reported in a separate paper with a clear reference to the PHETHAS-1 trial.

### Sample size

The sample size estimation is based on a minimal clinical important difference of 0.2 m/sec ^34^ between changes in gait speed among participants with highest performed exercise dose compared to participants with smallest performed exercise dose. Based on results from a pilot study leading up to this trial, we expect a maximal difference of 4 hours in performed exercise dose (total Time under tension summary dose) during the 7-week intervention period between participants with highest and lowest exercise compliance. Also based on the pilot study, a SD of 1.06 hours for exercise dose and 0.16 m/sec for change in gait speed were used. The power is set at 0.90 to increase the power for secondary analyses, and with a 0.05 level of significance. Based on the above, the required sample size is estimated to be 88 participants.

### Recruitment

**Figure 1.**
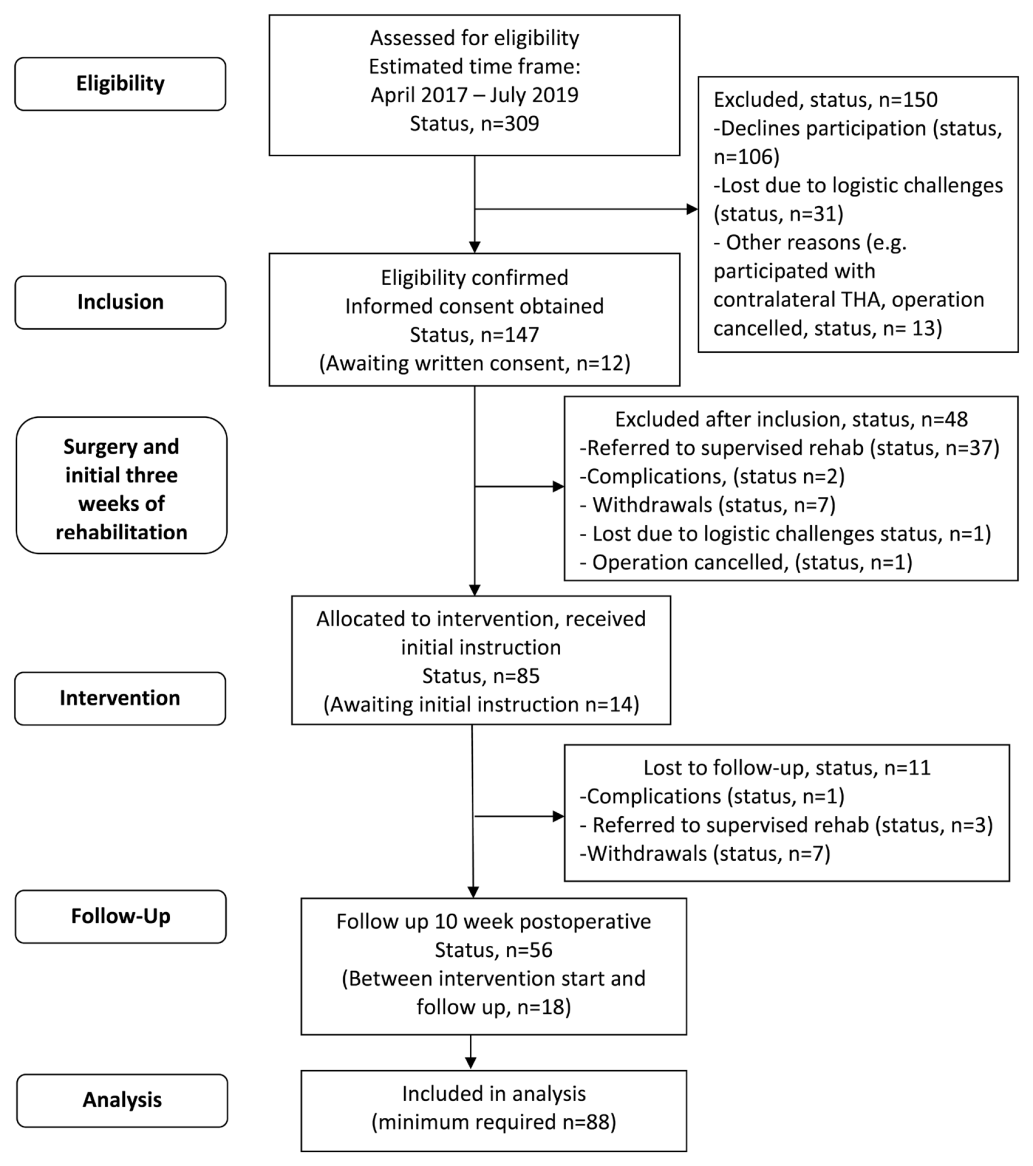
Estimated participant flow.

The basis for recruitment makes the trial highly feasible due to the approximately 800 elective THA procedures performed annually at the Elective Surgery Centre. As there may be more eligible participants per day than for whom there is available equipment (BandCizers and ActivPAL sensors), we restrict inclusion by including consecutive participants from random sections of the department. That is, patients examined and booked for surgery in pre-specified clinics in the outpatient department. Patients are allocated to the specific clinics in the department by a secretary at random and with no influence from any personnel involved in the study. The estimated inclusion rate is approximately one to two participants per week; please see estimated participant flow and current recruitment status in Figure 1.

### Blinding

The outcome assessors will be blinded to exercise compliance-data. Moreover, we will inform the participants that we measure how they perform their exercises and not how much they exercise or what the study hypothesis is. This is done with the purpose of minimizing sensor-induced influence on compliance and to reduce expectation bias.

### Data collection methods

The elastic band sensor (BandCizer) automatically records and stores exercise data during elastic band exercises. It is a valid measure of date, time of day, number of repetitions and sets, total time-under-tension (TUT), and total single repetition TUT during commonly used home-based strength training exercises for the lower extremities ^14^. The 40-m fast-paced walk test measures performance-based function and is part of the recommended core set of tests to assess physical function in people diagnosed with hip or knee osteoarthritis by the Osteoarthritis Research Society International (OARSI) ^26, 27^. A high inter-tester reliability is shown (intraclass correlation coefficient (ICC) 0.95) in a population with hip osteoarthritis ^34^. The HOOS questionnaire measures patient-reported outcome in the subscales: symptoms, pain, ADL, function in sport and recreation and hip-related quality of life ^28^. HOOS is shown valid, responsible, and reliable (ICC >0.78) when evaluating patients undergoing THA ^35^. Hip muscle strength ^29^ and 30-s chair stand test will be conducted in accordance with previous published methods ^27, 29^ showing acceptable relative and absolute inter-rater reliability when used after THA (ICC 0.83-0.93 and SEM 7–10%) ^36^. General Self-Efficacy Scale is a 10-item validated questionnaire holding a scale assessing optimistic self-beliefs to cope with a variety of difficult demands in life, scored between 1–4 points without any defined cut-off point ^30^. ActivPal movement-sensors measures physical activity as time spent in the sit/lie position (X-axis), standing (Y-axis) and walking (Z-axis). It has been validated in several studies in healthy adults ^37^ and in older adults with a hip fracture ^38, 39^.

Data collection will continue for participants who discontinue their training. Data collection will only be discontinued if participants explicitly withdraw from the study or any major events or diseases prevent the outcome assessments. If participants do not attend their scheduled follow ups, they will be contacted and offered a new time.

### Data management

Raw data from the Bandcizer will be uploaded to a secure online database using a tablet or smartphone. Here, the investigator will be able to access and analyze data and extract the following variables; date and number of training sessions, number of repetitions, time under tension for each repetition and total time under tension for each training session. Data from the outcome measurement will be double entered in EpiData 3.1 using anonymous coding with ID numbers and relevant range checks for data values to minimize typing errors. Completed data collection forms will be stored in a locked cabinet at Silkeborg Regional Hospital. Electronic data files will be stored on a secured hospital server with access requiring personal login. The linkage between ID numbers and personal identification data (e.g. civil registration number, name, address) will be stored as an electronic file as described above.

### Statistical methods

All the planned analyses are listed in Table 3.

**Table 3.** Variables, measures and methods of analysis.

Variable/outcome	Hypothesis	Outcome measure (unit, scale)	Methods of analysis
Demographic variables	Descriptive statistics, no hypothesis	Age, gender, height, weight, ASA status	Summary statistics
Supplementary descriptive variables	Descriptive statistics, no hypothesis	Prosthesis type, prior total joint replacement, length of hospital stay and self-efficacy (prior to surgery and baseline)	Summary statistics
Performed exercise dose of elastic band resistance training (exposure)	Descriptive statistics, no hypothesis	Time under tension summary dose/week and total (continuous) Number of exercise sessions/ week and total (number, continuous)	Summary statistics
1. Primary analysis			
Gait speed – measured by 40-m fast-paced walk test	Dose-response relationship	Change in score from baseline to follow up (meters/second, continuous)	(Described in more detail in section 2.12.1) Scatterplots of outcome variables and exercise dose variables will be used to suggest starting model structures and possible more complex alternatives. The analysis will depend on the specific relationship between change in gait speed and the exercise dose variable. Multiple models will be fitted and evaluated by R-squared values to identify the models that fit data the best. 1) The first model will be fitted as a fixed increase in outcome, based on exercise dose-change done by linear regression modelling. 2) If necessary, more complex regression, such as polynomial relationship and other nonlinear structures, will also be evaluated. 3) In the case that none of the models seem to fit the data, a linear regression model with a categorical variable based on intervals of the exercise dose variable will be fitted. If the measure at baseline correlates with the change, baseline measure will be adjusted for. The effect of possible confounding variables will be examined and, if having an effect, the confounder(s) will be adjusted for.
2. Secondary analyses			
HOOS ADL	Dose-response relationship	Change in score from baseline to follow up (points, continuous)	Similar to the primary analysis
HOOS symptoms, pain, QOL	Dose-response relationship	Change in score from baseline to follow up (points, continuous)	Summary statistics for the secondary outcomes will be presented as means with CIs or medians with IQRs within each of the compliance quartiles, and as graphical representation of these values as well.
30-s chair stand test		Change in score from baseline to follow up (repetitions, ordinal/continuous scale)	
Hip muscle strength (isometric)		Change in score from baseline to follow up (Nm/kg), continuous scale)	
3. Exploratory analyses			
Exercise compliance	Exploratory investigation of association. No hypothesis	Time under tension summary dose/week and total (continuous) Number of exercise sessions/ week and total (number, continuous)	Uni-variable regression models. Independent variables will be: pain flares (in the first two weeks of intervention and in the entire intervention period), HOOS pain (baseline), motivation to perform exercises, belief in effect of exercises, self-belief in compliance to exercising, satisfaction with rehabilitation exercise, physical activity (mean upright time/day and mean number of steps/day) and self-efficacy (baseline)
Physical activity level	Exploratory investigation of association. No hypothesis	Mean upright time per day (hours, continuous) and steps per day (numbers, continuous)	Uni-variable regression models. Independent variables will be: pain flares (first two weeks of intervention), HOOS pain (baseline), motivation to perform exercises, self-belief in compliance to exercising, and self-efficacy (baseline).
Result of the operation	Descriptive statistics, no hypothesis	Patient reported rating of perceived result after surgery (ordinal scale)	Data will be presented for each HOOS subscale (pain, symptoms, ADL, QOL) and gait speed. Data will be presented both for each response category, and for the subgroup of patients, who answered "excellent", "very good" or "good" data. This subgroup is considered to be reporting a hip-specific acceptable symptom state. Data will be presented by mean score with 95 % confidence intervals (CI) or median and inter quartile range (IQR). Furthermore, for each exercise dose quartile, the percentage of patients in each response category, will be presented (graphically). Finally, HOOS cut points for PASS will be estimated by the mean score or mean change approach
Change in hip problems	Descriptive statistics, no hypothesis	Patient reported rating of percieved problems in the operated hip now compared to before surgery (ordinal scale)	In each response category, the change in score from baseline to follow-up will be presented for each HOOS subscale (pain, symptoms, ADL, QOL) and gait speed. Data will be presented by mean with 95 % confidence intervals (CI) or median and inter quartile range (IQR). Furthermore, HOOS cut points for MCII will be estimated.
4. Other pre-specified outcomes			
Pain after exercise sessions	Descriptive statistics, no hypothesis	Change in pain per exercise session (score, continuous) Pain flare (increase in pain of ≥20 mm) during the first 14 days of the intervention (number, continuous) Pain flare for the entire intervention (number, continuous)	Summary statistics
Motivation to perform prescribed exercises	Descriptive statistics, no hypothesis	Motivation to perform exercises (ordinal scale) Belief in effect of exercises Self-belief in compliance to exercise	Summary statistics
Evaluation of prescribed exercises	Descriptive statistics, no hypothesis	Satisfaction with rehabilitation exercises Compliance with bandCizer Study-related change of exercise adherence	Summary statistics
Adverse events	Descriptive statistics, no hypothesis	Hip dislocation, infection, fracture, wound seepage, acute myocardial infarction, deep venous thrombosis, readmission, other)	Summary statistics

Descriptive analyses will be performed for demographic variables, supplementary descriptive variables, adverse events, motivation to perform prescribed exercises, evaluation of prescribed exercises and pain after exercise sessions (change in pain and pain flares). Data will be presented as means with 95% confidence intervals (CI) or medians with inter quartile ranges (IQR) for continues variables and as frequencies with percentages for categorical variables.

### Primary analysis

Initially, scatterplots of outcome variables and exercise dose variables will be used to suggest starting model structures and possible more complex alternatives. The structures of the models used for the dose-response analysis will depend on the specific relationship between change in gait speed and the exercise dose variable. Because of this, and not having any prior knowledge of the structure of the relationship, multiple models will be fitted and evaluated by R-squared values to identify the models that fit data the best. As a starting point, the first model will be fitted as a fixed increase in outcome, based on exercise dose-change done by linear regression modelling. If necessary, more complex regression such as polynomial relationship and other nonlinear structures will also be evaluated.

In the case that none of the models seem to fit the data, a linear regression model with a categorical variable based on intervals of the exercise dose variable will be fitted. This model does not provide a direct dose response relationship but provides an estimate of the association between the outcome variable and the exercise dose variable within the specific intervals.

“Regression to the mean” may be present and will be evaluated by the correlation between the change and the measure at baseline. If regression to the mean is believed to be present for an outcome, the models of the outcome will additional include the baseline measure to adjust for regression to the mean.

Possible confounding variables (self-efficacy (baseline), physical activity (during intervention), and gait speed (baseline)) will also be included in the models. The confounding effect of each variable will be examined by comparison of dose response estimates in models with and without the confounder. If there is no relevant change between the estimates of the models, the confounder will be excluded from the model. Normality assumptions in the models are evaluated by QQ-plots

### Secondary analyses

For the dose response relationship between change in HOOS ADL, the analysis will be similar to the analysis for change in gait speed outlined above.

The relationship between exercise compliance and HOOS subscales (symptoms, pain, quality of life), 30-s chair stand test and hip muscle strength will be presented as means with CIs or medians with IQRs within each of the compliance quartiles, as well as graphical representation of these values.

### Exploratory analyses

To better understand what may relate to how patients comply with prescribed rehabilitation exercise after THA, we will investigate how different variables relate to exercise compliance (dependent variables: time under tension summary dose and total number of exercise sessions), using uni-variable modelling. Independent variables will be: pain flares (first two weeks of intervention), pain flares (entire intervention period), HOOS pain (baseline), motivation to perform exercises, belief in effect of exercises, self-belief in compliance to exercising, satisfaction with rehabilitation exercise, physical activity (mean upright time/day and mean number of steps/day) and self-efficacy (baseline).

To better understand what may relate to how physically active patients are after a THA, we will investigate how different variables relate to physical activity (dependent variables: mean upright time/day and mean number of steps/day), using univariate modelling. Independent variables will be: pain flares (first two weeks of intervention), HOOS pain (baseline), motivation to perform exercises, self-belief in compliance to exercising, and self-efficacy (baseline).

In the analysis of "result of the operation", the change in score from baseline to follow-up will be presented for each HOOS subscale (pain, symptoms, ADL, QOL) and gait speed. Data will be presented both for each response category of the anchor question, and for the subgroup of patients, who answered "excellent", "very good" or "good" data. This subgroup is considered to be reporting a hip-specific acceptable symptom state. Data will be presented by means with 95% CI or medians and interquartile ranges (IQR). In each response category of the question for "change in hip problems", the change in score from baseline to follow-up will be presented for each HOOS subscale (pain, symptoms, ADL, QOL) and gait speed. Data will be presented by mean scores with 95% CI or median and inter quartile range (IQR).

Furthermore, for each exercise dose quartile, the percentage of patients in each response category of the questions for "result of the operation" and "change in hip problems", will be presented graphically. Finally, HOOS cut points for PASS and MCII will be estimated by the mean score or mean change approach ^40^.

### Handling of missing data

Based on the importance of the Bandcizer data, we have planned for how to use the data if we are not able to extract it as planned. Thus, we prioritize to use the Bandcizer data the following way depending on what is possible:

1) As planned with a valid total time-under-tension (TUT) estimate.

2) If 1) is not possible, we will use the total number of repetitions as an estimate of the total exercise dose.

3) If 1) and 2) is not possible, we will use the total number of days with performed exercise as an estimate of the total exercise dose.

We will report the proportion of missing data, e.g. self-reported non-compliance in Bandcizer use or invalid Bandcizer sensor-data. Furthermore, we will perform a sensitivity analysis using data on total exercise dose (days with exercise) from the exercise diary. This will inform if there is a comparable dose-response relationship when analyzing self-reported compliance data compared to objectively measured data (Bandcizer). The two types of data yield a risk of different types of bias. Self-reported exercise dose is often over-estimated and the Bandcizer data may induce problems with missing data as described above which could led to an underestimation of exercise-dose.

Missing items within the HOOS and General Self-efficacy scale will be handled as recommended in the guidelines (HOOS: <50% missing items in each subscale is accepted, self-efficacy: ≤3 missing items is accepted). Concerning ActivPal data, a minimum of four days of data collection will be accepted as sufficient to calculate min/day upright time and steps/day ^41^. In situations where participants have to stop the physical tests due to pain, the data from the best performance are used no matter if the pre-defined number of repetitions is reached. It is noted if tests are interrupted due to pain to be able to perform sensitivity analysis if appropriate. If participants are lost to follow up (despite the before-mentioned efforts to keep every participant in the trial) they will be excluded from the analyses that include change scores. We will not use last-observation-carried-forward or other imputation procedures on exposure (exercise dose), as we aim to investigate relationships between actually performed exercise dose and changes in post-operative outcomes. However, on possible confounding variables that are included in the model, we will use multiple imputation if needed to retain the sample size of n=88 in the analysis. Models used in the imputation will include the remaining confounders with measures as predictors.

### Data monitoring

Since the study involves no major changes to current practice it is not deemed necessary to establish a data monitoring committee or perform any interim analyses. Likewise, no provisions for post-trail care will be made.

## Discussion

This trial will add knowledge concerning the preliminary efficacy of home-based rehabilitation using elastic band exercises based on the relationship between performed exercise dose and outcomes after THA. We believe this is the first trial to do so, since earlier attempts have not used objective measurement of exercise dose as in the present trial. In an observational cohort study, Zech *et al.* found no significant associations between the exercise therapy intensity or duration and improvements in patient reported function, pain, and stiffness ^42^. However, the exercise dose was dependent on the participants´ health insurance as well as individual conditions and the physiotherapist's decision, which likely induces a risk of bias by indication.

The essential need from a clinical perspective is to be able to prescribe evidence-based exercise programs after THA. Despite the growing number of studies, a recent systematic review that included 20 studies concludes that insufficient therapeutic validity and potentially high risk of bias in the included studies limit the ability to assess the effectiveness of exercise after THA ^43^.

The new knowledge from the present study can potentially identify whether the dose of performed home-based exercise is related to changes in post-operative outcomes after THA. It will give insight concerning the potential influence from other factors than exercise, such as general physical activity and self-efficacy. Furthermore, the embedded qualitative study will give insight to perceived motivation and barriers to perform the prescribed exercise as well as to resuming general physical activities. The results from both the quantitative and qualitative study are expected to be useful in optimizing current practice; however, the results will also be used to plan, power and execute a randomized controlled trial that compares the effectiveness of rehabilitation exercises to no rehabilitation exercises (just resuming general physical activities).

### Strength and limitations

The strengths of this study include the objectively measured exercise dose, the standardized and thoroughly described intervention and the inclusion of outcome variables at all levels in the International Classification of Function, Disability and Health (ICF). We chose gait speed measured by the 40-m fast-paced walk test as the primary outcome. Walking ability is considered the most important function to improve by patients undergoing THA surgery ^25^, and the 40-m fast-paced walk test is part of the core set of functional tests to include in clinical trials in patients with osteoarthritis in hip or knee recommended by OARSI ^26^. An important candidate for the choice of primary outcome for clinical research has been suggested to be a patient-reported one ^44^. Nevertheless, we chose a performance-based measure as the primary, as we were concerned about ceiling effects on patient reported outcomes that measures function and pain, such as the HOOS questionnaire after THA ^45^.

Multiple factors can potentially affect exercise compliance; therefore, we include measurements of physical activity and self-efficacy. Also, it is not known which outcomes that is most susceptible to exercise dose which is why we include a broad range of different outcome types to be able to explore potential dose-response relationships.

Blinding of participants in randomized exercise trials are often impossible, in the present study we seek to blind the participants to the specific focus om exercise dose, they are just told that we measure "the way they exercise". Hypothesis blinding is considered a design strength when blinding of participants regarding treatment is not possible ^46^. Furthermore, we blind the outcome assessor in the sense that they are not allowed to see the exercise diary or BandCizer data prior to the outcome assessment.

## Trial status

The trial began recruiting participants in April 2017. After a period with slow inclusion, the inclusion rate is back at 1–2 participants per week, thus, inclusion is expected to be completed in July 2019. See current status on participant flow in Figure 1.

This paper is based on protocol version 5, March 8, 2019.

## Declarations

### Research ethics approval

The Ethics Committee of Central Denmark Region accepted initiation of the study and reviewed the study as non-notifiable (Inquiry 270/2017). The study was approved by the Danish Data Protection Agency (ref. no: 1-16-02-589-15).

### Informed consent

Trained Research staff (nurse or physiotherapist) will provide presentation of comprehensible information about the research to potential participants, confirmation that they understand the research, and assurance that their agreement to participate is voluntary. Potential participants will also receive information sheets. They will be offered deliberation time and, subsequently, written consent will be obtained from those who choose to participate. The informed consent document is available as *Extended data* ^20^.

### Confidentiality

All records that contain names or other personal identifiers, such as informed consent forms, will be stored separately from study records identified by code number to protect confidentiality before, during, and after the trial.

### Future availability of trial data

The principal investigator, as well as all co-authors, will have access to the full dataset as needed. A fully anonymized dataset and statistical analysis code will be made available for the scientific journal reviewing the manuscript within six months in line with the recent proposal from the International Committee of Medical Journal Editors (ICMJE) ^47^.

### Dissemination policy

Results from the trial will be published in international, scientific peer-reviewed journals, no matter the trial outcome. The results will also be presented at relevant scientific conferences and symposiums. Authorships will be allocated according to the ICMJE recommendations. The following papers are planned:

1. Pragmatic Home-Based Exercise after Total Hip Arthroplasty – Silkeborg (PHETHAS-1): Results from a prospective cohort study.
2. Motivation and barriers to perform home-based exercise after Total Hip Arthroplasty – a qualitative embedded study within the PHETHAS-1 trial.

## Data availability

### Underlying data

No underlying data are associated with this article.

### Extended data

Figshare: PHETHAS-1 protocol. https://doi.org/10.6084/m9.figshare.8256014 ^20^.

This project contains the following extended data:

- WHO Trial Registration Data Set_PHETHAS.docx
- Consent document.pdf
- Managing pains associated with exercise.docx (pain management guide)
- PHETHAS Interview guide_english (PHETHAS-2 interview guide, translated into English)
- Questionnaire_ Motivation to perform exercises.docx
- Questionnaire_Evaluation of prescribed exercises.docx
- CERT Checklist.docx
- TIDieR Checklist.docx

Extended data are available under the terms of the Creative Commons Zero “No rights reserved” data waiver (CC0 1.0 Public domain dedication).

### Reporting guidelines

Figshare: SPIRIT checklist for “Pragmatic Home-Based Exercise after Total Hip Arthroplasty - Silkeborg: Protocol for a prospective cohort study (PHETHAS-1)”. https://doi.org/10.6084/m9.figshare.8256014 ^20^.

## Footnotes

^1^ Abbreviations: ADL, Activities of Daily Living; CI, Confidence Interval; HOOS, Hip disability and Osteoarthritis Outcome Score, ICMJE, International Committee of Medical Journal Editors; IQR, Inter Quartile Range; NRS, Numeric Rating Scale; THA, Total Hip Arthroplasty; PHETHAS, Pragmatic Home-Based Exercise after Total Hip Arthroplasty – Silkeborg; RM, Repetition Maximum; STROBE, STrengthening the Reporting of OBservational studies in Epidemiology; TIDieR, Template for Intervention Description and Replication; TUT, time-under-tension; VAS, Visual Analogue Scale; WHO, World Health Organisation
