# Supplementary material for: Pragmatic Home-Based Exercise after Total Hip Arthroplasty - Silkeborg: Protocol for a prospective cohort study (PHETHAS-1)
Source: F1000Res. 2019 Oct 14;8:965. Originally published 2019 Jun 25. [Version 2] (PMC6694449)
Supplement: Peer review history

# Peer review: Reviewer response for version 1

**DOI:** 10.5256/f1000research.21458.r50849

**Published:** 2019-08-30

**Authors:** Martin Stevens, Sjoukje Bouma

**Affiliations:** 1 Department of Orthopedic Surgery, University Medical Center Groningen (UMCG), University of Groningen, Groningen, The Netherlands; 2 Department of Orthopedic Surgery, University Medical Center Groningen (UMCG), University of Groningen, Groningen, The Netherlands

This is a very well-written and interesting study protocol focusing on the effectiveness of home-based rehabilitation following total hip arthroplasty. Specific strengths of the protocol are the very detailed description of the study design and the content of the home-based exercise intervention in particular, the objectively quantified exercise dose and the combination of quantitative and qualitative methods which will be used to evaluate the home-based exercise intervention. However, a few items were encountered while reading the manuscript which the authors might reflect upon:

- The different aspects of the home-based exercise intervention are described in great detail (Table 2), which enhances the understanding and replicability of the intervention. However, it is not entirely clear why these specific four strengthening exercises (hip abduction, flexion, extension with elastic band resistance and sit-to-stand) and the intervention duration of 7 weeks were chosen. Please elaborate on the rationale for these choices regarding the design on the home-based exercise intervention (e.g. based on guidelines or recommendations?).
- On page 4, it is stated that participants will be advised to comply with the recommendations on physical activity from the Danish Health and Medicines Authority. Could you give a (short) description of these recommendations?
- On page 4, it is described that all participants will receive a thorough instruction in the strengthening exercises at the baseline measurement and that face-to-face meetings among physiotherapists will be held to reinforce similar treatment administration. Will patients also be monitored during the intervention period to check whether they are still performing the strengthening exercises according to the received instructions?
- Outcome measures (1): 24-hour physical activity level will only be determined at the start of the intervention. Why will this outcome measure not be repeated at the end of the intervention as well?
- Outcome measures (2): in contrast to hip abductor and hip flexor muscle strength, hip extensor muscle strength is not specified as secondary outcome measure. However, hip extensor muscle strength is one of the four strengthening exercises of the interventions. Could you clarify why this will not be evaluated as secondary outcome?
- Self-efficacy (1): to measure self-efficacy, the general self-efficacy scale will be used. However, it has been stated that the concept of self-efficacy is most useful when it is measured for a particular behavior in a specific context (e.g. postoperative rehabilitation following total hip arthroplasty) (Bandura, 1997) ^1^. Could you elaborate on the decision to include a general self-efficacy scale instead of a more context-specific self-efficacy scale (e.g. The Self-Efficacy For Rehabilitation Outcome Scale, Waldrop *et al.* 2001) ^2^?
- Self-efficacy (2): Table 1 shows that self-efficacy will be measured both prior to surgery (admission) and 3 weeks after surgery (baseline measurement). Literature indicates that short-term postoperative self-efficacy seems to be a better predictor of long-term outcome following total joint arthroplasty than preoperative self-efficacy (Scheek *et al*. 2007) ^3^. You seem to incorporate this finding by only including the postoperative self-efficacy as possible confounding variable in the statistical analyses. What will be the additional value of including the preoperative self-efficacy measurement in your study design?

We confirm that we have read this submission and believe that we have an appropriate level of expertise to confirm that it is of an acceptable scientific standard.

## References

[1] : Self-efficacy: The exercise of control.1997;

[2] : Self-efficacy, optimism, health competence, and recovery from orthopedic surgery. *Journal of Counseling Psychology*.2001;48(2) : 10.1037/0022-0167.48.2.233 233-238 10.1037/0022-0167.48.2.233

[3] : Preoperative or postoperative self-efficacy: which is a better predictor of outcome after total hip or knee arthroplasty?. *Patient Educ Couns*.2007;66(1) : 10.1016/j.pec.2006.10.012 92-9 10.1016/j.pec.2006.10.012 17223004

# Response

**Published:** 2019-10-09

**Authors:** Lone Ramer Mikkelsen

**Affiliations:** Silkeborg Regional Hospital, Denmark

**Response to Reviewer 3**

*Thank you for your positive evaluation. Below we address your comments one by one.*

**Reviewer 3, question 1: **The different aspects of the home-based exercise intervention are described in great detail (Table 2), which enhances the understanding and replicability of the intervention. However, it is not entirely clear why these specific four strengthening exercises (hip abduction, flexion, extension with elastic band resistance and sit-to-stand) and the intervention duration of 7 weeks were chosen. Please elaborate on the rationale for these choices regarding the design on the home-based exercise intervention (e.g. based on guidelines or recommendations?).

**Response:** The type of exercise and the dose reflects our current practice which we want to evaluate (pragmatic approach). To our knowledge, there is currently not evidence to support a specific type of exercise after THA as more efficient than others, as we mention in the introduction: "It has also been difficult to demonstrate clear superiority with relevant effect size of one type of rehabilitation exercise over another for performance-based or self-reported function outcomes ^8^^, ^^9^". In a previous study, we have shown that an intervention with strengthening exercises using elastic band (more or less similar exercises to the present study) was comparable to supervised exercise in strength training machines (reference 1). The focus on strengthening exercise is based on the documented deficits in muscle strength after THA (references 2 and 4 in the manuscript). The rationale for this focus is also that adequate muscle strength is a requisite to re-establish a normal, symmetric gait pattern.

**Changes made.** Text added in the section "Intervention": The exercises in the present trial are comparable to the control intervention in a previous study from our department where we compared usual care (home-based exercise using elastic band resistance) to supervised progressive resistance training in machines and found comparable effects (Mikkelsen *et al*, 2014, ref 1 in response to reviewer).

**Reviewer 3, question 2: **On page 4, it is stated that participants will be advised to comply with the recommendations on physical activity from the Danish Health and Medicines Authority. Could you give a (short) description of these recommendations?

**Response:** We agree and have added a description.

**Changes made:** Text added in the section "Intervention": (≥30 minutes/day of physical activity with moderate intensity + 20 minutes twice a week of physical activity with high intensity)

**Reviewer 3, question 3: **On page 4, it is described that all participants will receive a thorough instruction in the strengthening exercises at the baseline measurement and that face-to-face meetings among physiotherapists will be held to reinforce similar treatment administration. Will patients also be monitored during the intervention period to check whether they are still performing the strengthening exercises according to the received instructions?

**Response:** We only monitor on compliance concerning exercise dose (the Bandcizer data and the exercise diary). We have no monitoring of movement quality in the exercise. This could be relevant from a clinical point of view, however it would be difficult to standardize for use in research.

No changes made

**Reviewer 3, question 4: **Outcome measures (1): 24-hour physical activity level will only be determined at the start of the intervention. Why will this outcome measure not be repeated at the end of the intervention as well?

**Response:** It would be ideal to have objectively measured activity data from the entire intervention period; however that would be very time consuming for both patients and researchers. We consider physical activity as a potential confounder on the association between exercise dose and gait speed. We can not guarantee that the one week with activPal measurements is representative for the entire intervention period. However, 7 days is a typical time frame when measuring activity level and (reference 2). We could have repeated the measurement on ActivPal in the end or middle of the intervention. However, it would require an extra effort by both patients and staff because the patients are not at the hospital during that period. We already ask the patients to collect a lot of data when using the Bandcizer at every exercise session as well as the ActivPal the first week. All things considered, we decided that one week of objectively measured activity, as a proxy for the general activity level during the intervention period, should be sufficient.

No changes made

**Reviewer 3, question 5: **Outcome measures (2): in contrast to hip abductor and hip flexor muscle strength, hip extensor muscle strength is not specified as secondary outcome measure. However, hip extensor muscle strength is one of the four strengthening exercises of the interventions. Could you clarify why this will not be evaluated as secondary outcome?

**Response:** Patients after total hip replacement are often limited in their range of motion in hip extension impeding the performance of hip extensions strength test in prone position. We have not been able to find a valid way to measure hip extension strength with handheld dynamometer in these patients which is why we chose to focus solely on hip abduction and flexion.

No changes made

**Reviewer 3, question 6: **Self-efficacy (1): to measure self-efficacy, the general self-efficacy scale will be used. However, it has been stated that the concept of self-efficacy is most useful when it is measured for a particular behavior in a specific context (e.g. postoperative rehabilitation following total hip arthroplasty) (Bandura, 1997) ^1^. Could you elaborate on the decision to include a general self-efficacy scale instead of a more context-specific self-efficacy scale (e.g. The Self-Efficacy For Rehabilitation Outcome Scale, Waldrop *et al.* 2001) ^2^?

**Response:** We agree that a context-specific self-efficacy scale would have been preferable. However, we have not been able to identify a Danish version of the The Self-Efficacy For Rehabilitation Outcome Scale, thus it would require a translation process prior to initiating the study. An advantage of using the general self-efficacy scale is to be able to compare our findings to previous studies on THA patients where that scale has often been used (e.g. reference 3-5).

No changes made

**Reviewer 3, question 7: **Self-efficacy (2): Table 1 shows that self-efficacy will be measured both prior to surgery (admission) and 3 weeks after surgery (baseline measurement). Literature indicates that short-term postoperative self-efficacy seems to be a better predictor of long-term outcome following total joint arthroplasty than preoperative self-efficacy (Scheek *et al*. 2007) ^3^. You seem to incorporate this finding by only including the postoperative self-efficacy as possible confounding variable in the statistical analyses. What will be the additional value of including the preoperative self-efficacy measurement in your study design?

**Response:** Preoperative self-efficacy is only included as a descriptive variable and not included in the analysis. The argument for this is, exactly as stated in your comment, that post-operative self-efficacy seems to be a better predictor than preoperative measurement.The preoperative measurement will increase the possibility to compare the study population to other studies. Also it will give a descriptive indication to whether self-efficacy is a constant variable or if it changes from pre- to postoperative in our sample of patients.

No changes made

References in response to Reviewer 3

1. Mikkelsen LR, Mechlenburg I, Søballe K, *et al*.: Effect of early supervised progressive resistance training compared to unsupervised home-based exercise after fast-track total hip replacement applied to patients with preoperative functional limitations. A single-blinded randomised controlled trial. *Osteoarthritis Cartilage.* 2014 Dec;22(12):2051-8
2. Migueles JH, Cadenas-Sanchez C, Ekelund U, et al.: Accelerometer Data Collection and Processing Criteria to Assess Physical Activity and Other Outcomes: A Systematic Review and Practical Considerations. *Sports Med.* 2017;47(9):1821–1845. 
3. Brembo E, Kapstad H, Van Dulmen S, *et al.*: Role of self-efficacy and social support in short-term recovery after total hip replacement: a prospective cohort study. *Health Qual Life Outcomes*. 2017 Apr 11;15(1):68.
4. Olsson LE, Hansson E, Ekman I. Evaluation of person-centred care after hip replacement-a controlled before and after study on the effects of fear of movement and self-efficacy compared to standard care. *BMC Nurs.* 2016 Sep 9;15(1):53. 
5. Jørgensen LB, Mikkelsen LR, Noe BB, *et al.*: The psychosocial effect of web-based information in fast-track surgery. *Health Informatics J.* 2017 Dec;23(4):304-318.

# Peer review: Reviewer response for version 1

**DOI:** 10.5256/f1000research.21458.r50851

**Published:** 2019-08-14

**Authors:** Kristi Elisabeth Heiberg, Vigdis Bruun-Olsen

**Affiliations:** 1 Department of Medical Research, Bærum Hospital, Vestre Viken Hospital Trust, Drammen, Norway; 2 Oslo Metropolitan University, Oslo, Norway

This is a generally well-written, interesting, and well-designed study, on an important topic. Both from a therapeutic and health-economic perspective it is important to make the patients with THA self-reliant in their exercise management. We also consider the qualitative part of the proposed trial as an important contribution to understand the patients’ barriers and motivation for performing home-based exercises after THA.

 However, we have some comments to the authors:

- It is pointed out that there is some evidence to indicate that rehabilitation exercise may be superior to no or very little rehabilitation exercise after THA (Introduction section). Can you clarify the construct ‘rehabilitation exercise’?
- The rationale for using elastic band seems a bit unclear in the section of introduction. How can the authors argue for open kinetic chain strength training with elastic band compared to functional task-oriented training? Is there any prior research indicating the effectiveness of strength training in an open kinetic chain?
- The term ‘pragmatic’ should be clarified (page 3).  
- Participants: We consider the inclusion criteria very wide and unspecific (18 years and OA?). There seems to be few exclusion criteria. Why were patients with stroke, other neurological diseases, drug abuse etc., not excluded from the study?
- As walking ability is considered the most important function to improve by patients undergoing THA, why were activities of walking not included in the training? What is the rationale and link between elastic band training and improvements in walking?
- Regarding dose-response; how do you deal with the eventual problem of registration of little activity with the use of the elastic band and a high general weight-bearing activity, such as walking, without registration? In total, this can be considered as a large dose of activity that is not registered. The ActivePAL is only applied for one week.
- The planned statistical analyses are well described and seem to be adequate.

We confirm that we have read this submission and believe that we have an appropriate level of expertise to confirm that it is of an acceptable scientific standard.

# Response

**Published:** 2019-10-09

**Authors:** Lone Ramer Mikkelsen

**Affiliations:** Silkeborg Regional Hospital, Denmark

**Response to Reviewer 2**

*Thank you for your positive evaluation. Below we address your comments one by one.*

**Reviewer 2, question 1:** It is pointed out that there is some evidence to indicate that rehabilitation exercise may be superior to no or very little rehabilitation exercise after THA (Introduction section). Can you clarify the construct ‘rehabilitation exercise’?

**Response:** We define rehabilitation exercise as: "A regimen or plan of physical activities designed and prescribed for specific therapeutic goals. Its purpose is to restore normal musculoskeletal function or to reduce pain caused by diseases or injuries."

This definition is synonymous to the MESH term "Exercise therapy" as defined in the PubMed MeSH database.

**Changes made:** Definition added in the section "Intervention".

**Reviewer 2, question 2: **The rationale for using elastic band seems a bit unclear in the section of introduction. How can the authors argue for open kinetic chain strength training with elastic band compared to functional task-oriented training? Is there any prior research indicating the effectiveness of strength training in an open kinetic chain?

**Response:** The construct functional task-oriented training is difficult to define. To our knowledge, there is currently not evidence to support a specific type of exercise after THA as more efficient than others, as we mention in the introduction: "It has also been difficult to demonstrate clear superiority with relevant effect size of one type of rehabilitation exercise over another for performance-based or self-reported function outcomes ^8^^, ^^9^". In a previous study, we have shown that an intervention with open kinetic chain exercises using elastic band (more or less similar elastic band exercises as in the present study) was comparable to closed kinetic chain exercise in strength training machines (reference 1). In that study both groups was also advised to be physically active (e.g. by walking). In the current study we also include the task-oriented, closed kinetic chain exercise "sit to stand" as well as advise to perform daily gait training.

**Changes made:** Text added in the section "Intervention": The exercises in the present trial is comparable to the control intervention in a previous study from our department where we compared usual care (home-based exercise using elastic band resistance) to supervised progressive resistance training in machines and found comparable effects (Mikkelsen *et al*, 2014, ref 1 in response to reviewer).

The patients are recommended to perform daily walking with increasing distance during their rehabilitation.

**Reviewer 2, question 3: **The term ‘pragmatic’ should be clarified (page 3).

**Response:** By pragmatic study we mean that the study reflects real life for the involved trial stakeholders. In this study this is for instance reflected by the type and dose of exercise which reflects our current practice.

**Changes made:** Definition added in the section "Study design".

**Reviewer 2, question 4: **Participants: We consider the inclusion criteria very wide and unspecific (18 years and OA?). There seems to be few exclusion criteria. Why were patients with stroke, other neurological diseases, drug abuse etc., not excluded from the study?

**Response:** As this study is designed to reflect current practice (pragmatic approach) we used a minimum of exclusion criteria. There is a continuum between explanatory trials (many exclusion criteria, undertaken in an idealized setting, to give the intervention under evaluation its best chance to demonstrate a beneficial effect) and pragmatic trials (undertaken in the “real world” and with usual care and is intended to help support a decision on whether to deliver an intervention). For elaboration see PRagmatic Explanatory Continuum Indicator Summary (PRECIS-2) ( https://www.precis-2.org/) and reference 2.

No changes made

**Reviewer 2, question 5: **As walking ability is considered the most important function to improve by patients undergoing THA, why were activities of walking not included in the training? What is the rationale and link between elastic band training and improvements in walking?

**Response:** Daily walking and increasing general physical activity is recommended to the participants. The focus on elastic band exercise is based on the documented deficits in muscle strength after THA (references 2 and 4 in the manuscript). The rationale for this focus is that adequate muscle strength is a requisite to re-establish a normal, symmetric gait pattern. For many of the patients this is after years of limping due to pain. In the current study we combine specific strengthening exercise (elastic band) with functional task exercise (sit-to-stand) and functional training through physical activities – primarily walking, but also resumption of activities preferred by the patients which is encouraged.  

**Changes made:** Text added in the section "Intervention": The patients are recommended to perform daily walking with increasing distance during their rehabilitation. They are advised to gradually increase their general activity level after the operation to comply with the recommendations on physical activity from the Danish Health and Medicines Authority (≥30 minutes/day of physical activity with moderate intensity + 20 minutes twice a week of physical activity with high intensity).

**Reviewer 2, question 6: **Regarding dose-response; how do you deal with the eventual problem of registration of little activity with the use of the elastic band and a high general weight-bearing activity, such as walking, without registration? In total, this can be considered as a large dose of activity that is not registered. The ActivePAL is only applied for one week.

**Response: **We agree that it is crucial to measure not only the patients' effort during the specific exercises, but also their other physical activities. This is why we include the ActivPal measurement as well as the exercise diary where the patients also register their physical activities. It would be ideal to have objectively measured activity data from the entire intervention period; however that would be very time consuming for both patients and researchers. We consider the physical activity as a potential confounder on the association between exercise dose and gait speed. We can not guarantee that the one week with activPal measurements is representative for the entire intervention period. However, 7 days is a typical time frame when measuring activity level and (ref 40 in manuscript). We could have repeated the measurement on ActivPal in the end or middle of the intervention. However, it would require an extra effort by both patients and staff because the patients are not at the hospital during that period. We already ask the patients to collect a lot of data using the Bandcizer at every exercise session as well as the ActivPal the first week. All things considered, we decided that one week of objectively measured activity as a proxy for the general activity level during the intervention period should be sufficient.

No changes made.

References in response to Reviewer 2

 

1. Mikkelsen LR, Mechlenburg I, Søballe K, *et al*.: Effect of early supervised progressive resistance training compared to unsupervised home-based exercise after fast-track total hip replacement applied to patients with preoperative functional limitations. A single-blinded randomised controlled trial. *Osteoarthritis Cartilage.* 2014 Dec;22(12):2051-8
2. Loudon K, Treweek S, Sullivan F, *et al.*: The PRECIS-2 tool: designing trials that are fit for purpose. *BMJ.* 2015 May 8;350:h2147.

# Peer review: Reviewer response for version 1

**DOI:** 10.5256/f1000research.21458.r50853

**Published:** 2019-08-02

**Authors:** Dana L. Judd

**Affiliations:** 1 Physical Therapy Program, University of Colorado, Aurora, CO, USA

This protocol paper details a prospective cohort study which will investigate the initial efficacy of home-based exercise program following total hip arthroplasty (THA). The authors present strong rationale for performing the study, in particular citing evidence regarding the deficits in strength and function following THA as well as the lack of consensus regarding rehabilitation following THA surgery.

 The study design is described in great detail, and the proposed methods will adequately answer the research question. The strengths of the study design include objective monitoring of exercise adherence, the standardization of the exercise protocol, and the combination of validated strength measures and functional performance outcome measures. The choice of exercises for the intervention target common deficits following THA, and the choice of a gait outcome as a primary outcome measure will represent an important functional outcome for patients. A unique aspect of this proposed trial is the proposed qualitative investigation into patients' perceived barriers to and motivations for participating in the proposed exercise program. Not only will this have potential to inform future trials, but may provide important information from the patients' point of view to better inform clinical practice and home exercise prescription after THA. 

 There are also items the authors may wish to consider. First, although there are proposed training and meetings to ensure correct instruction by therapists to patients, the authors may consider including a way to objectively report fidelity data of the therapists to describe how well they adhered to the protocol. Second, due to the importance of collecting the exercise adherence data, is there a plan to handle missing data from the Bandcizer sensors? Or perhaps a way to ensure data is being collected over the weeks of intervention as expected and a plan for troubleshooting. Finally, although the choice of exercises for the protocol aligns well with common impairments following THA, it is possible the few specific exercises and proposed dosing may not yield the expected changes in gait speed without the inclusion of gait-specific training. 

 Overall, this is a well written manuscript describing a well-designed study and the results should be valuable to the literature regarding rehabilitation after THA.

I confirm that I have read this submission and believe that I have an appropriate level of expertise to confirm that it is of an acceptable scientific standard.

# Response

**Published:** 2019-10-09

**Authors:** Lone Ramer Mikkelsen

**Affiliations:** Silkeborg Regional Hospital, Denmark

**Response to Reviewer 1**

*Thank you for your positive evaluation. Below we address your items for consideration one by one.*

**Reviewer 1, question 1**: First, although there are proposed training and meetings to ensure correct instruction by therapists to patients, the authors may consider including a way to objectively report fidelity data of the therapists to describe how well they adhered to the protocol.

**Response:** We agree that it would be relevant to include an objective measure of fidelity in the exercise instructions. We did not consider this when we started the trial, and at the present time, inclusion is almost completed. What we did consider, however, was to continuously standardize the instructions through regular meetings and discussions with the therapists administering the intervention. We feel this reflects clinical practice and, hence, the pragmatic approach that we wanted for the trial. The main discussion points at these meetings, until now, have primarily concerned adaptations to the exercise program when the patients experience pain during or after exercise. That is why we have developed the pain management guide which is published as extended data. Furthermore, we use ongoing reminders of the importance of thorough instructions in performing exercise to failure.

No changes made in manuscript

**Reviewer 1, question 2:** Second, due to the importance of collecting the exercise adherence data, is there a plan to handle missing data from the Bandcizer sensors? Or perhaps a way to ensure data is being collected over the weeks of intervention as expected and a plan for troubleshooting

**Response:** This is a good point and based on previous experiences with this technology, which is still under development, we expect that such a plan will be important. We have added plans for handling missing data and troubleshooting. To prevent discontinued use of the Bandcizer sensor during exercise, we inform the patients of the importance of using the Bandcizer sensors in every single exercise session. To estimate if this is accomplished, we ask the patients at the 10 week follow up visit: "How often was the BandCizer sensor attached to your elastic exercise band when exercising?"" (options: Every time, most of the time about half the time, a few times, never). This is described in "Extended data"

**Changes made:** Text added in the section "Handling of missing data": Based on the importance of the Bandcizer data, we have made a plan for how to use the data if we are not able to extract the data as planned. Thus, we prioritize to use the Bandcizer data the following way depending on what is possible:

1) As planned with a valid total time-under-tension (TUT) estimate.

2) If 1) is not possible, we will use the total number of repetitions as an estimate of the total exercise dose.

3) If 1) and 2) is not possible, we will use the total number of days with performed exercise as an estimate of the total exercise dose.

We will report the proportion of missing data, e.g. self-reported non-compliance in Bandcizer use or invalid Bandcizer sensor-data. Furthermore, we will perform a sensitivity analysis using data on total exercise dose (days with exercise) from the exercise diary. This will inform if there is a comparable dose-response relationship when analyzing self-reported compliance data compared to objectively measured data (Bandcizer). The two types of data yield a risk of different types of bias. Self-reported exercise dose is often over-estimated and the Bandcizer data may induce problems with missing data as described above which could led to an underestimation of exercise-dose.

We will not use last-observation-carried-forward or other imputation procedures on exposure (exercise dose), as we aim to investigate relationships between actually performed exercise dose and changes in post-operative outcomes. However, on possible confounding variables that are included in the model, we will use multiple imputation if needed to retain the sample size of n=88 in the analysis. The models used in the imputation will include the remaining confounders with measures as predictors.

**Reviewer 1, question 3:** Finally, although the choice of exercises for the protocol aligns well with common impairments following THA, it is possible the few specific exercises and proposed dosing may not yield the expected changes in gait speed without the inclusion of gait-specific training. 

**Response:** The type of exercise and the dose reflects our current practice which we want to evaluate. To our knowledge this is not a smaller exercise dose compared to clinical practice many places. It is still unclear if the prescribed exercises, and exercise dose, is sufficient to improve gait speed. However, it is more comprehensive than the "control group" intervention in our previous study where supervised resistance training was compared to home-based exercise and found comparable in effect (reference 1). If the instructions are followed correctly, the total time-under-tension is 10 min per session, 3-4 times per week equaling 30-40 min/week during 7 weeks. We could have included some gait-specific exercises, but instead we chose, in a pragmatic approach, to comply with the current clinical practice at our institution. All patients were encouraged to be physically active (e.g. by walking) as per the recommendations from the Danish Health and Medicines Authority. They were recommended to perform daily walking with increasing distance during their rehabilitation.

**Changes made:** Text added in the section "Intervention": The exercises in the present trial is comparable to the control intervention in a previous study from our department where we compared usual care (home-based exercise using elastic band resistance) to supervised progressive resistance training in machines and found comparable effects (Mikkelsen *et al*, 2014, ref 1 in response to reviewer).

The patients are recommended to perform daily walking with increasing distance during their rehabilitation. They are advised to gradually increase their general activity level after the operation to comply with the recommendations on physical activity from the Danish Health and Medicines Authority (≥30 minutes/day of physical activity with moderate intensity + 20 minutes twice a week of physical activity with high intensity).

Furthermore Table 2 has been corrected since we discovered a mistake concerning time-under-tension for each exercise. The corrected values are 2 seconds concentric (instead of 1 in the first version) + 1 second isometric and 2 seconds eccentric, equaling 150 sec/exercise/set.

Reference in response to Reviewer 1

 

1. Mikkelsen LR, Mechlenburg I, Søballe K, *et al*.: Effect of early supervised progressive resistance training compared to unsupervised home-based exercise after fast-track total hip replacement applied to patients with preoperative functional limitations. A single-blinded randomised controlled trial. *Osteoarthritis Cartilage.* 2014 Dec;22(12):2051-8
